# Diabetes but Not Insulin Increases the Risk of Lung Cancer: A Taiwanese Population-Based Study

**DOI:** 10.1371/journal.pone.0101553

**Published:** 2014-07-03

**Authors:** Chin-Hsiao Tseng

**Affiliations:** 1 Department of Internal Medicine, National Taiwan University College of Medicine, Taipei, Taiwan; 2 Division of Endocrinology and Metabolism, Department of Internal Medicine, National Taiwan University Hospital, Taipei, Taiwan; 3 Division of Environmental Health and Occupational Medicine of the National Health Research Institutes, Taipei, Taiwan; Massachusetts Eye & Ear Infirmary, Harvard Medical School, United States of America

## Abstract

**Background:**

The trend of lung cancer incidence in Taiwan is unknown, and the association between type 2 diabetes/insulin use and lung cancer is rarely studied.

**Methods:**

The trends of lung cancer incidence in 1979–2007 in the Taiwanese general population were calculated. A random sample of 1,000,000 subjects covered by the National Health Insurance in 2005 was recruited. A total of 494,002 men and 502,948 women and without lung cancer were followed for the annual cumulative incidence of lung cancer in 2005, with calculation of the risk ratios between diabetic and non-diabetic subjects. Logistic regression estimated the adjusted odds ratios for risk factors.

**Results:**

The trends increased significantly in both sexes (*P*<0.0001). The sex-specific annual cumulative incidence increased with age in either the diabetic or non-diabetic subjects, but the risk ratios attenuated with age. In logistic regressions, diabetes was associated with a significantly higher risk, with odds ratios (95% confidence interval) for diabetes duration <1, 1–3, 3–5 and ≥5 years versus non-diabetes of 2.189 (1.498-3.200), 1.420 (1.014-1.988), 1.545 (1.132-2.109), and 1.329 (1.063-1.660), respectively. Such an association was not related to a higher detection with chest X-ray examination. Insulin use and medications including oral anti-diabetic drugs, statin, fibrate, and anti-hypertensive agents were not significantly associated with lung cancer. Age, male sex, and chronic obstructive pulmonary disease were positively; but dyslipidemia, stroke and higher socioeconomic status were negatively associated with lung cancer.

**Conclusions:**

Diabetes is significantly associated with a higher risk of lung cancer, but insulin use does not increase the risk.

## Introduction

Lung cancer is the most common cancer around the world [1]. In Taiwan, lung cancer is currently the third most common cancer in either sex, but it ranks as the second and first cancerous killer in men and women, respectively [2]. There has been no report on the secular trends of lung cancer incidence in Taiwan over the past decades.

Diabetic patients are prone to develop cancer involving pancreas, liver, breast, colorectum, bladder and endometrium [3]. However, the association between diabetes and lung cancer is rarely studied. Studies conducted in western countries showed contradictory results. In earlier studies from UK [4] and the USA [5], diabetes was not associated with the risk of lung cancer. However, two recent studies, from Denmark [6] and Sweden [7], respectively, suggested a significantly higher risk of lung cancer in the diabetic patients.

Insulin use has been implicated as a risk factor for cancer [3]. However, whether insulin use would increase the risk of lung cancer is an issue that has not been extensively investigated. In the UK study, insulin therapy with or without oral anti-diabetic agents was not associated with lung cancer [4], but in the Danish study both insulin users and non-users have significantly higher risk of lung cancer [6]. None of the studies have compared the risk between insulin users and non-users.

Because diabetes affects hundreds of millions of people worldwide and insulin is commonly used for the control of blood glucose in patients with type 2 diabetes mellitus (T2DM), it is important to elucidate the link between T2DM/insulin use and lung cancer. The purpose of the present study was to evaluate 1) the trends of lung cancer incidence in the Taiwanese general population from 1979 to 2007 by using the Taiwan Cancer Registry database; and 2) the association between T2DM/insulin use and lung cancer by analyzing the National Health Insurance (NHI) reimbursement database.

## Materials and Methods

### Trends of lung cancer incidence in 1979–2007

The study was approved by the National Health Research Institutes of Taiwan (Registered number 99274). The trends of both crude and age-standardized (to the 2000 World Health Organization population) lung cancer incidence in 1979–2007 in the general population were first calculated from the released database (available online) of the Taiwan Cancer Registry [8]. The population-based registry was established in 1979 and supported by the Department of Health of Taiwan. It is an ongoing registry including all hospitals with more than 50 beds in Taiwan. Newly diagnosed cases of cancer should be reported and the completeness and accuracy are evaluated each year. The proportion of histologically verified cases of lung cancer was 92.7% [2].

### Annual incidence of lung cancer in 2005 and related risk factors

According to the Ministry of Interior, Taiwan, in 2005, >98.0% of the Taiwanese population (22,770,383: 11,562,440 men and 11,207,943 women) was covered by the NHI. A random sample of 1,000,000 people insured by the NHI in 2005 was created by the National Health Research Institutes for academic research. The National Health Research Institutes is the only organization approved, as per local regulations, for conducting sampling of a representative sample of the whole population for the year 2005 with a predetermined sample size of 1,000,000 individuals. The reimbursement databases of these sampled individuals were retrieved and could be provided for academic research after approval. The identification information was scrambled for the protection of the privacy of the sampled individuals. The reimbursement databases from 1996 onward were available. Sex, birth date, medications, and diagnostic codes based on the *International Classification of Diseases, Ninth Revision, Clinical Modification* (ICD-9-CM) were retrieved for analyses in this study. Diabetes was coded 250.1-250.9 and lung cancer, 162.

**Figure 1:**
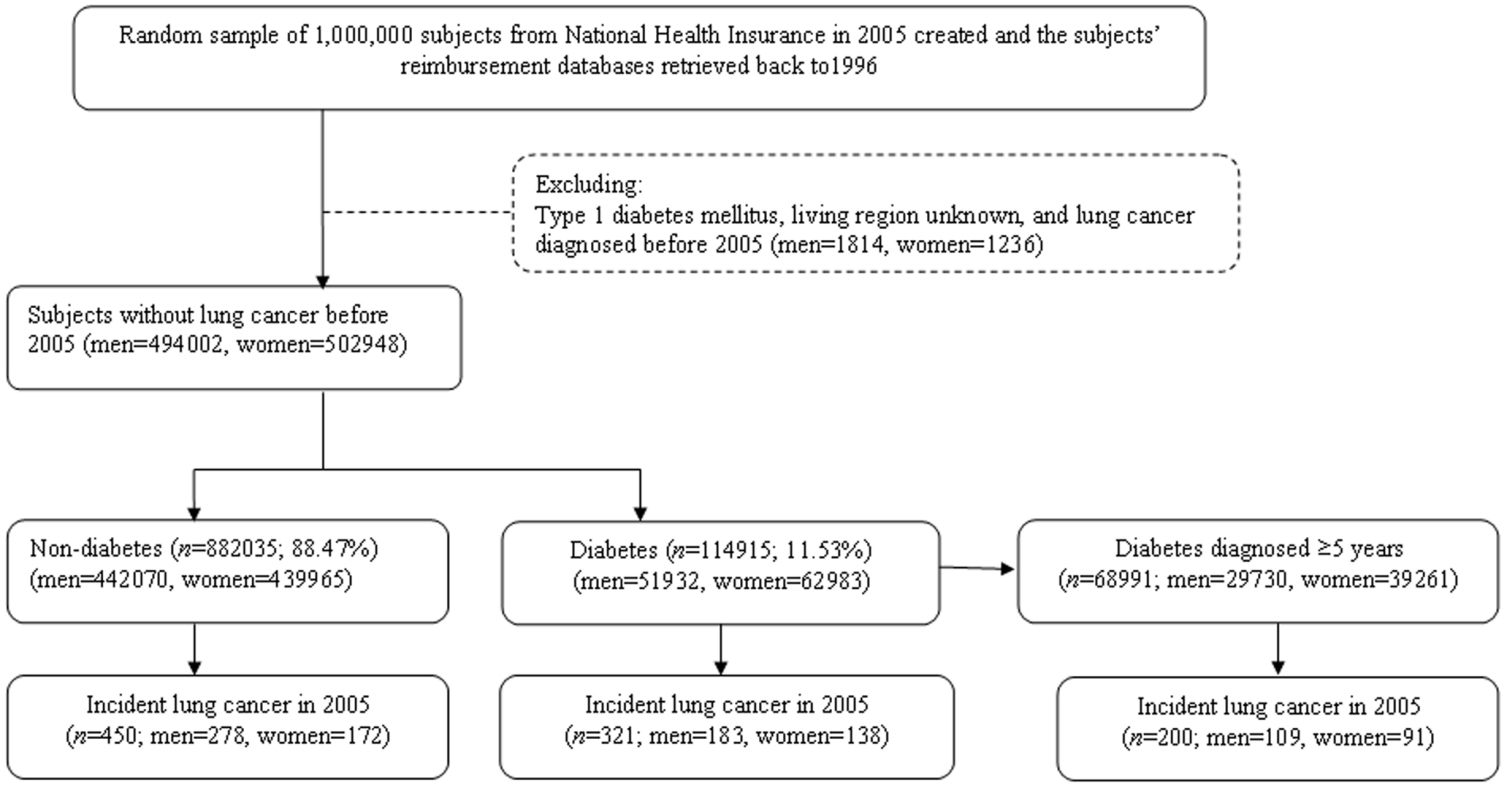
Flowchart showing the procedures in the calculation of the annual incidence of lung cancer in 2005 in Taiwan.

Figure 1 shows a flowchart used for selecting cases in this study. After excluding subjects with type 1 diabetes (in Taiwan, patients with type 1 diabetes were issued a ‘Severe Morbidity Card’ after certified diagnosis), subjects for whom the living region was not known, and subjects diagnosed with lung cancer before 2005, 494,002 men and 502,948 women were recruited for the calculation of the annual incidence of lung cancer in 2005.

### Statistical analyses

Analyses were conducted using the SAS statistical software, version 9.1 (SAS Institute, Cary, NC). *P*<0.05 was considered statistically significant.

Linear regression evaluated whether the trends of lung cancer incidence in 1979–2007 in the general population changed significantly, where the incidence was the dependent and the calendar year the independent variable.

The age- and sex-specific annual cumulative incidences of lung cancer in 2005 in diabetic and non-diabetic subjects were calculated for all ages and age <40, 40–64, 65–74 and ≥75 years. The numerator was the number of patients with a first diagnosis of lung cancer within 2005; and the denominator was the number of insurants in that specific group. The risk ratio between diabetic and non-diabetic subjects was calculated and the 95% confidence interval (CI) estimated by Taylor series approximation [9]. To minimize the possibility that diabetes might be caused by lung cancer, lag time sensitivity analyses were performed by excluding patients with diabetes duration of <5 years.

Logistic regression calculated the adjusted odds ratios. Lung cancer was the dependent variable and the independent variables included age (<40, 40–64, 65–74 and ≥75 years), diabetes duration (non-diabetes, <1, 1–3, 3–5 and ≥5 years), chest X-ray examination (CXR), comorbidities, medications, living region and occupation. CXR is the most commonly used screening tool for lung cancer [10], [11] and was included in the models to control for the potential detection bias through screening. The comorbidities (ICD-9-CM codes) included hypertension (401-405), chronic obstructive pulmonary disease (COPD, 490-496, a surrogate for smoking), stroke (430–438), nephropathy (580–589), ischemic heart disease (410–414), peripheral arterial disease (250.7, 785.4, 443.81, 440–448), eye disease (250.5, 362.0, 369, 366.41, 365.44), obesity (278) and dyslipidemia (272.0–272.4). Medications included statin, fibrate, angiotensin-converting enzyme inhibitor and/or angiotensin receptor blocker, calcium channel blocker, sulfonylurea, metformin, insulin, acarbose, pioglitazone and rosiglitazone. Insulin use was categorized as yes versus no (model I), and according to the duration of its use of <5 years and ≥5 years versus non-users (model II). Comorbidities and medications were counted only as they appeared before 2005 to assure temporal correctness of cause and effect (lung cancer). The NHI insurants were classified according to occupation and this served as a surrogate for socioeconomic status. The living region served as a surrogate for geographical distribution of some environmental exposure. Occupation was categorized as I: civil servants, teachers, employees of governmental or private business, professionals and technicians; II: people without particular employers, self-employed or seamen, III: farmers or fishermen; and IV: low-income families supported by social welfare or veterans. Living region was categorized as Taipei, Northern, Central, Southern and Kao-Ping/Eastern. The regressions were performed for all ages and for age ≥40 years, separately.

## Results

**Figure 2:**
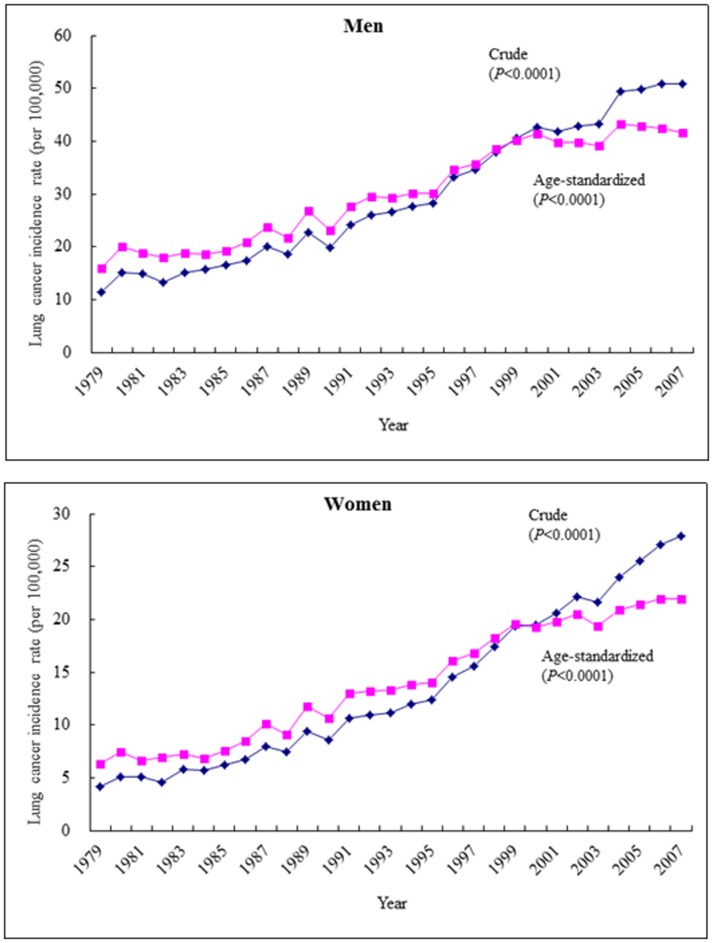
Trends of lung cancer incidence in the general population of Taiwan from 1979 to 2007 (diamonds: crude rates, squares: age-standardized rates using the 2000 World Health Organization population as referent).

Figure 2 shows the crude and age-standardized incidence trends in 1979–2007. Both are increasing significantly in either sex (*P*<0.0001).

Table 1 shows the cumulative incidences and the risk ratios between the diabetic and non-diabetic subjects. The cumulative incidence markedly increased with age in either the diabetic or non-diabetic subjects. Risk ratio analysis showed that diabetic patients had a higher risk than non-diabetic subjects in all age groups, though the risk ratios attenuated with increasing age and would not be significant in the older age groups in either sex.

**Table 1:** Age-sex-specific rates (per 100,000) of annual incidence of lung cancer in 2005 and risk ratios between diabetic and non-diabetic subjects.

Sex/Rate/Risk ratio	Age (years)
	All ages	<40	40–64	65–74	≥75
**Men**					
**Diabetes of any duration**					
**Diabetic men**					
*n* of lung cancer	183	2	40	53	88
* n* of diabetic men	51934	6978	26361	9883	8712
Rate in diabetic men	352.37	28.66	151.74	536.27	1010.10
**Non-diabetic men**					
* n* of lung cancer	278	13	92	62	111
* n* of non-diabetic men	442080	282848	128347	17755	13130
Rate in non-diabetic men	62.88	4.60	71.68	349.20	845.39
**Risk ratio (95% CI)**	5.60 (4.65–6.75)	6.24 (1.41–27.63)	2.12 (1.46–3.07)	1.54 (1.07–2.21)	1.19 (0.90–1.58)
**Excluding diabetes diagnosed <5 years**					
**Diabetic men**					
* n* of lung cancer	109	2	24	29	54
* n* of diabetic men	29730	3221	14029	6438	6042
Rate in diabetic men	366.63	62.09	171.07	450.45	893.74
**Non-diabetic men**					
* n* of lung cancer	278	13	92	62	111
* n* of non-diabetic men	442080	282848	128347	17755	13130
Rate in non-diabetic men	62.88	4.60	71.68	349.20	845.39
**Risk ratio (95% CI)**	5.83 (4.67–7.27)	13.51 (3.05–59.84)	2.39 (1.52–3.74)	1.29 (0.83–2.00)	1.06 (0.76–1.46)
**Women**					
**Diabetes of any duration**					
**Diabetic women**					
* n* of lung cancer	138	3	37	48	50
* n* of diabetic women	62986	10170	30488	13121	9207
Rate in diabetic women	219.10	29.50	121.36	365.83	543.07
**Non-diabetic women**					
* n* of lung cancer	172	19	77	31	45
* n* of non-diabetic women	439975	286159	126040	16660	11116
Rate in non-diabetic women	39.09	6.64	61.09	186.07	404.82
**Risk ratio (95% CI)**	5.60 (4.48–7.01)	4.44 (1.31–15.01)	1.99 (1.34–2.94)	1.97 (1.25–3.09)	1.34 (0.90–2.00)
**Excluding diabetes diagnosed <5 years**					
**Diabetic women**					
* n* of lung cancer	91	1	29	26	35
* n* of diabetic women	39263	5298	18081	9158	6726
Rate in diabetic women	231.77	18.88	160.39	283.90	520.37
**Non-diabetic women**					
* n* of lung cancer	172	19	77	31	45
* n* of non-diabetic women	439975	286159	126040	16660	11116
Rate in non-diabetic women	39.09	6.64	61.09	186.07	404.82
**Risk ratio (95% CI)**	5.93 (4.60–7.64)	2.84 (0.38–21.23)	2.63 (1.71–4.02)	1.53 (0.91–2.57)	1.29 (0.83–2.00)

CI = confidence interval

Table 2 shows the results of the logistic regressions for all ages and for age ≥40 years, respectively. The results were similar in models I and II. Only the odds ratios for age, sex, diabetes duration and insulin use are shown for the models conducted for age ≥40 years because the odds ratios for other variables are similar to those observed in the analyses for all ages.

**Table 2:** Mutually-adjusted odds ratios for lung cancer derived from incident cases in 2005 for all ages and age ≥40 years.

Variables	Interpretation	Model I	Model II
		OR	95% CI	*P* value	OR	95% CI	*P* value
**All ages**							
Age	40–64 years vs. <40 years	9.548	(6.718–13.571)	<0.0001	9.548	(6.718–13.572)	<0.0001
	65–74 years vs. <40 years	29.089	(19.941–42.433)	<0.0001	29.089	(19.942–42.434)	<0.0001
	≥75 years vs. <40 years	51.981	(35.588–75.923)	<0.0001	51.985	(35.591–75.929)	<0.0001
Sex	Men vs. Women	1.452	(1.254–1.681)	<0.0001	1.452	(1.254–1.681)	<0.0001
Diabetes duration	<1 year vs. Non-diabetes	2.189	(1.498–3.200)	<0.0001	2.189	(1.498–3.200)	<0.0001
	1–3 years vs. Non-diabetes	1.420	(1.014–1.988)	0.0414	1.420	(1.014–1.989)	0.0413
	3–5 years vs. Non-diabetes	1.545	(1.132–2.109)	0.0062	1.545	(1.132–2.110)	0.0061
	≥5 years vs. Non-diabetes	1.329	(1.063–1.660)	0.0124	1.329	(1.063–1.660)	0.0125
Insulin	Yes vs. No	0.808	(0.492–1.327)	0.3992	-	-	-
	<5 years vs. Non-user	-	-	-	0.882	(0.216–3.606)	0.8609
	≥5 years vs. Non-user	-	-	-	0.800	(0.476–1.345)	0.3999
Chest X-ray	Yes vs. No	3.355	(2.717–4.143)	<0.0001	3.355	(2.717–4.143)	<0.0001
Hypertension	Yes vs. No	0.991	(0.818–1.202)	0.9302	0.991	(0.817–1.202)	0.9297
COPD	Yes vs. No	1.204	(1.032–1.404)	0.0182	1.204	(1.032–1.404)	0.0183
Stroke	Yes vs. No	0.774	(0.639–0.936)	0.0083	0.774	(0.639–0.936)	0.0083
Nephropathy	Yes vs. No	0.859	(0.693–1.064)	0.1632	0.859	(0.693–1.064)	0.1632
IHD	Yes vs. No	1.120	(0.944–1.328)	0.1943	1.120	(0.944–1.328)	0.1943
PAD	Yes vs. No	1.003	(0.800–1.258)	0.9795	1.003	(0.800–1.258)	0.9799
Eye disease	Yes vs. No	0.965	(0.663–1.407)	0.8548	0.965	(0.662–1.406)	0.8517
Obesity	Yes vs. No	1.113	(0.526–2.353)	0.7803	1.113	(0.526–2.354)	0.7799
Dyslipidemia	Yes vs. No	0.813	(0.667–0.991)	0.0404	0.813	(0.667–0.991)	0.0404
Statin	Yes vs. No	1.018	(0.792–1.307)	0.8918	1.017	(0.792–1.307)	0.8923
Fibrate	Yes vs. No	0.853	(0.655–1.110)	0.2359	0.853	(0.656–1.110)	0.2362
ACEI/ARB	Yes vs. No	1.064	(0.847–1.337)	0.5949	1.064	(0.847–1.337)	0.5943
CCB	Yes vs. No	0.989	(0.786–1.244)	0.9246	0.989	(0.786–1.244)	0.9243
Sulfonylurea	Yes vs. No	1.097	(0.799–1.506)	0.5680	1.097	(0.799–1.506)	0.5677
Metformin	Yes vs. No	1.020	(0.731–1.423)	0.9063	1.020	(0.731–1.423)	0.9064
Acarbose	Yes vs. No	1.016	(0.628–1.646)	0.9470	1.017	(0.628–1.646)	0.9460
Pioglitazone	Yes vs. No	1.602	(0.794–3.234)	0.1885	1.603	(0.794–3.235)	0.1882
Rosiglitazone	Yes vs. No	0.927	(0.559–1.538)	0.7706	0.928	(0.559–1.539)	0.7720
Living region	Northern vs. Taipei	0.899	(0.703–1.150)	0.3987	0.899	(0.703–1.150)	0.3984
	Central vs. Taipei	1.040	(0.838–1.292)	0.7195	1.040	(0.838–1.292)	0.7198
	Southern vs. Taipei	1.150	(0.918–1.442)	0.2250	1.150	(0.917–1.441)	0.2254
	Kao-Ping/Eastern vs. Taipei	1.213	(0.990–1.486)	0.0625	1.213	(0.990–1.485)	0.0628
Occupation	II vs. I	1.299	(1.026–1.644)	0.0297	1.299	(1.026–1.644)	0.0297
	III vs. I	1.280	(1.037–1.580)	0.0213	1.280	(1.037–1.580)	0.0213
	IV vs. I	1.424	(1.166–1.739)	0.0005	1.424	(1.166–1.739)	0.0005
**Age ≥40 years** *							
Age	65–74 years vs. 40–64 years	3.071	(2.488–3.789)	<0.0001	3.071	(2.488–3.789)	<0.0001
	≥75 years vs. 40–64 years	5.514	(4.467–6.806)	<0.0001	5.514	(4.467–6.806)	<0.0001
Sex	Men vs. Women	1.512	(1.300, 1.759)	<0.0001	1.512	(1.300–1.759)	<0.0001
Diabetes duration	<1 year vs. Non-diabetes	2.131	(1.448–3.135)	0.0001	2.131	(1.448–3.135)	0.0001
	1–3 years vs. Non-diabetes	1.421	(1.014–1.991)	0.0414	1.421	(1.014–1.992)	0.0412
	3–5 years vs. Non-diabetes	1.513	(1.105–2.073)	0.0099	1.513	(1.105–2.073)	0.0099
	≥5 years vs. Non-diabetes	1.300	(1.038–1.628)	0.0225	1.300	(1.037–1.628)	0.0226
Insulin	Yes vs. No	0.815	(0.496–1.339)	0.4192	-	-	-
	<5 years vs. Non-user	-	-	-	0.893	(0.218–3.652)	0.8743
	≥5 years vs. Non-user	-	-	-	0.807	(0.480–1.356)	0.4182

Insulin use is categorized as yes versus no (Model I), and according to the duration of its use (Model II).

OR: odds ratio, CI: confidence interval, COPD: chronic obstructive pulmonary disease, IHD: ischemic heart disease, PAD: peripheral arterial disease, ACEI: angiotensin-converting enzyme inhibitor, ARB: angiotensin receptor blocker, CCB: calcium channel blocker.

Refer to Materials and Methods for the categories of occupation.

*For age ≥40 years, only the odds ratios for age, sex, diabetes duration and insulin are shown. The odds ratios for other variables are not remarkably different from those seen in the analyses for all ages.

## Discussion

The trends of lung cancer were increasing significantly in 1979–2007 (Figure 2) and diabetes was associated with an increased risk at any duration (Table 2), with the highest risk ratio observed in the youngest age of <40 years (Table 1). However, insulin use was not associated with a significant change in the risk of lung cancer (Table 2).

Recently two retrospective cohort analyses using the NHI database to evaluate the association between diabetes and lung cancer in Taiwan have been reported [12], [13]. The study by Lai et al. selected a group of new-onset diabetic patients aged ≥20 years (*n* = 19,624) from 2000–2005 and a group of non-diabetic patients matched on age and sex at baseline (n = 78,496) and traced the incidence of lung cancer for a period of up to 9 years in these two groups [12]. They concluded that there was no association between diabetes and lung cancer. However, this study could possibly be under-powered without sufficient case numbers. Furthermore, they have neglected the high incidence of diabetes during the long duration of follow-up in the baseline non-diabetic group with a mean age of 56.5 years. Another study by Lee et al. followed 985,815 insurants with and without diabetes in 1997 and followed them from 1998 to 2009 [13]. They found that diabetes at baseline was associated with a significantly higher risk of lung cancer, with risk ratio (95% CI) of 1.54 (1.26–1.88) after adjustment for age, sex, hypertension, dyslipidemia and gout. This study also neglected the possible incidence of diabetes among the baseline non-diabetic group during the long duration of follow-up. Therefore, this study could have possibly underestimated the risk of lung cancer associated with diabetes.

The present study strengthened the link between diabetes and lung cancer; and supported a lack of association with insulin use. Diabetes was unlikely caused by lung cancer because diabetes diagnosed 5 years before lung cancer (Tables 1 and 2) can hardly be a consequence of the carcinogenic process. Detection bias was also unlikely because CXR had been adjusted in the analyses (Table 2).

Diabetes has been on the rise worldwide [14], especially in Asian countries [15]. In Taiwan diabetes prevalence increased from 5.0% in 1970 to 12.8% in 1996 in those aged ≥40 years [16]. The incidence of T2DM in Taiwan showed a significant increase of 1.8-fold from 1992 to 1996 [17]. If diabetes increases the risk of lung cancer, the incidence of lung cancer is expected to increase in the years to come, especially in Asian countries where diabetic patients outnumber those of the other continents [14], [15].

None of the medications including insulin would affect the risk of lung cancer (Table 2). Our previous studies suggested that insulin use may be associated with some forms of cancer but not all: insulin use is predictive for incidence of [18] but not mortality from [19] non-Hodgkin's lymphoma; it is associated with mortality from but not incidence of bladder cancer [20], [21], liver cancer [22], [23] and pancreatic cancer [24], [25]; and it is neither associated with the incidence of nor the mortality from prostate cancer [26]–[28] and gastric cancer [29], [30]. Putting together the findings of a lack of predictive power of insulin use on the mortality from lung cancer in our recent prospective 12-year follow-up study [31] and the lack of an association with the incidence of lung cancer in the present study (Table 2), the lung may also be one of those less sensitive organs. Therefore, some organs may be especially sensitive to exogenous insulin for cancer development, while others are not.

COPD and lung cancer share the same risk factor of smoking, and COPD is also significantly associated with a higher risk of lung cancer [1]. The observation that COPD significantly increased the risk of lung cancer (Table 2) strongly implied that smoking could be the common risk factor for both conditions; and that the findings of the present study was not likely biased without the adjustment of smoking.

It is interesting that stroke and dyslipidemia were associated with a significantly lower risk of lung cancer (Table 2). One explanation for the lower risk in patients with stroke is that these patients might have a higher mortality before they would have the opportunity to develop lung cancer. The lower risk in patients with dyslipidemia could not be due to the protective effects of the lipid-lowering drugs such as statin or fibrate, because both have been adjusted in the models (Table 2). One possibility is that dyslipidemia is strongly associated with cardiovascular diseases, which may develop at a younger age and lead to premature death. Therefore these patients may have a lower probability of developing lung cancer, which is more likely to occur after the age of 65 years (Table 1).

Although geographical distribution as indicated by the living region was not significantly associated with lung cancer, people with the highest socioeconomic status as indicated by occupation I did show a significantly lower risk while compared to the other groups (Table 2).

Some cases of lung cancer may have been misclassified in the study. However, such an occurrence was probably low because labeled diagnoses should be printed on all prescriptions handed out to patients in Taiwan. Mislabeling of a cancer diagnosis would not be acceptable to patients when they saw the diagnosis. Age, male sex and COPD are major risk factors for lung cancer [1]. They were also significantly associated with lung cancer here. Therefore the findings derived from the models are unlikely to be biased because these risk factors were also observed here (Table 2).

The present study was not able to evaluate the histological patterns or clinical stages of lung cancer. According to the Taiwan Cancer Registry, adenocarcinoma is the most common pathology representing 40.4% and 70.6% in men and women, respectively; followed by squamous cell carcinoma, representing 24.9% and 7.9%, respectively [2]. Therefore, a majority of the lung cancer reported here must be related either to adenocarcinoma or squamous cell carcinoma.

This study has several strengths. It is population-based with a large nationally representative sample. The database included outpatients and inpatients and we caught the diagnoses from both sources. Cancer is considered as a severe morbidity by the NHI and most medical co-payments can be waived. Therefore the detection rate would not tend to differ among different social classes. The use of medical record also reduced the potential bias related to self-reporting.

Limitations included a lack of actual measurement of confounders such as smoking, alcohol drinking, family history, lifestyle, diet, and genetic parameters. In addition, we did not have biochemical data for evaluating their impact. Finally, the follow-up interval is probably too short to plausibly account for the likely induction time needed between the onset of diabetes and the biological changes leading to lung cancer.

In summary, this study shows an increasing trend of lung cancer in Taiwan and a link between diabetes and lung cancer, which is more remarkable in the age of <40 years. Insulin or other medications are not, but COPD and lower socioeconomic status are significantly associated with lung cancer. The lower risk in patients with dyslipidemia and stroke awaits further investigation. Given that the population is aging, the incidence of lung cancer is increasing, and the incidence of T2DM is also increasing [17], the impact of diabetes-related lung cancer on the population should warrant public health attention.
